# Effect of Allicin and Artesunate Combination Treatment on Experimental Mice Infected with *Plasmodium berghei*

**DOI:** 10.1155/2022/7626618

**Published:** 2022-04-18

**Authors:** Sakaewan Ounjaijean, Voravuth Somsak

**Affiliations:** ^1^Research Institute for Health Sciences, Chiang Mai University, Chiang Mai 50200, Thailand; ^2^School of Allied Health Sciences, Walailak University, Nakhon Si Thammarat 80160, Thailand; ^3^Research Excellence Center for Innovation and Health Products, Walailak University, Nakhon Si Thammarat 80160, Thailand

## Abstract

Malaria is still a significant health problem in endemic countries and increases *Plasmodium* resistance to the available antimalarial drugs. Hence, this study aimed to investigate the antimalarial activity of allicin and its combination with artesunate (ART) against rodent malaria *Plasmodium berghei* ANKA (PbANKA) infected mice. Allicin was prepared in 20% Tween-80. Balb/c mice were inoculated intraperitoneally with 1×10^7^ PbANKA-infected erythrocytes and orally given by gavage with the chosen doses of 1, 10, 50, and 100 mg/kg of allicin and 1, 5, 10, and 20 mg/kg of ART once a day for 4 consecutive days. Effective dose 50 (ED_50_) of allicin and ART was subsequently investigated. Moreover, the combination (1 : 1) of allicin and ART at the doses of their respective ED_50_, ED_50_ 1/2, ED_50_ 1/4, and ED_50_ 1/8 was also carried out. The untreated control was given 20% Tween-80. The results showed that allicin presented a dose-dependent antimalarial activity with significance (*p* < 0.05). The ED_50_ values of allicin and ART were about 14 and 5 mg/kg, respectively. For combination, allicin and ART showed a synergistic effect at the combination doses of ED_50_, ED_50_ 1/2, and ED_50_ 1/4 with significantly (*p* < 0.01) prevented reduction of packed cell volume, bodyweight loss, rapid dropping of rectal temperature, and markedly prolonged mean survival time, compared with the untreated control and single treatment. It can be concluded that allicin exerted potential antimalarial activity in single and its combination with ART.

## 1. Introduction

Malaria is one of the serious public health problems in developing countries. In 2019, there were an estimated 229 million cases of malaria and 409000 malarial deaths, in which 94% of the cases occurred in sub-Saharan African [1]. Malaria in humans is caused by parasite in the genus *Plasmodium* and spread to people through infected female *Anopheles* mosquito bites. There are five species of human malaria parasites, including *Plasmodium falciparum*, *Plasmodium vivax, Plasmodium ovale, Plasmodium malariae*, and *Plasmodium knowlesi*, and 2 of these species, *P. falciparum* and *P. vivax*, pose the greatest threat [2]. Even though the effective malarial vaccine is the best control for infection and a small clinical trial testing vaccine against malaria has shown promising results, the researchers still need phase III trials to confirm the vaccine's efficacy and safety [3, 4]. Therefore, the most attention is currently focused on antimalarial drugs. There are only a limited number of available antimalarial drugs that can prevent or treat malaria such as quinine and its derivatives, chloroquine, and antifolate combination drugs. However, resistance of the antimalarial drug has emerged and implicated in the spread of malaria to new areas and the reemergence of malaria in areas where the disease had been eradicated [5]. Artemisinin-based combination therapies (ACTs) are now generally recommended by the World Health Organization as the first-line treatment for uncomplicated falciparum malaria in malarial endemic areas [6]. Unfortunately, it has been described that artemisinin resistance is found in the Greater Mekong Subregion of Southeast Asia, where it is associated with treatment failures for some ACTs [7, 8]. The finding and development of new, safe, and affordable antimalarial drugs are urgently needed.

Allicin (diallylthiosulfinate) is the most abundant sulfur-containing natural compound found in garlic (*Allium sativum L*.) with a broad spectrum of biological activities [9]. Allicin has been reported to have antibacterial and antifungal activities in a dose-dependent manner, including methicillin-resistant *Staphylococcus aureus* (MRSA) [10–12]. Furthermore, induction of cancer cell death and inhibition of cancer cell proliferation by allicin have also been described [13]. Allicin has also been described to have various health-promoting properties, such as free radical scavenging and antioxidant, anti-inflammation, antiparasitic, antidiabetic, lipid-lowering, hepatoprotective, cardioprotective, and neuroprotective activities [14]. Additionally, it has been revealed that allicin significantly inhibited sporozoite invasion of *P*. *berghei* and presented antimalarial activity in mice [15]. However, the effective dose of allicin and its combination with artesunate (ART) in malaria treatment has not yet been studied.

Since the experimental-based evidence for the antimalarial effect of combination treatment is found essential, this study was first designed to evaluate the antimalarial activity of allicin interaction with ART in experimental mice infected with rodent malaria parasite *P*. *berghei*.

## 2. Materials and Methods

### 2.1. Chemicals

Allicin, ART, and Tween-80 purchased from Sigma-Aldrich (Sigma-Aldrich, St. Louis, MO, USA) were used. All reagents were analytical grade. Before experiments, the chosen doses of allicin and ART were freshly prepared in 20% Tween-80 and administered orally by gavage. The untreated mice were administered only 20% Tween-80.

### 2.2. Experimental Animal

Healthy 6–8 weeks old male Balb/c mice, weighing 25–30 g, used throughout this study, were obtained from Nomura Siam International Co., Ltd., Bangkok, Thailand. They were acclimatized for one week at the animal room with a temperature of 22–25°C, 50–60% humidity, and 12 h light-dark cycle. Mice were allowed free access to a commercial pellet food and clean water ad libitum. All experiments associated animals were ratified and approved by the Animal Ethical Committee, Walailak University (WU-ACUC-65002).

### 2.3. *Plasmodium berghei*

*Plasmodium berghei* strain ANKA (PbANKA) provided by Malaria Research and Reference Reagent Resource Center (MR4; https://www.beiresources.org/About/MR4.aspx) was used in this study. PbANKA was maintained by mechanical serial passage of intraperitoneal (IP) inoculation of 1×10^7^ PbANKA-infected erythrocytes into Balb/c mice weekly. Parasite growth was daily monitored by microscopy of Giemsa-stained thin blood smear of 500–2000 erythrocytes (200–300 erythrocytes/field) using 100*x* immersion objective. Calculation of parasitemia was performed using the following formula.

(1) $$\begin{array}{c} \%\text{parasitemia}=\frac{\text{Number of parasitized erythrocytes}}{\text{ Number of total erythrocytes}}\times 100. \end{array}$$

Blood was then collected from the infected mice with 15–20% parasitemia by cardiac puncture under anesthesia in 200 U/ml of heparinized tubes. The collected blood was then diluted with phosphate buffer saline (PBS) to obtain 5×10^7^ PbANKA-infected erythrocytes, and 0.2 ml was injected into each mouse by IP inoculation.

### 2.4. Antimalarial Activity of Allicin against PbANKA

The antimalarial effect of allicin was conducted in PbANKA-infected mice using a standard 4-day suppressive test as previously described [16]. Healthy Balb/c mice were divided into 9 groups of 5 mice each and inoculated by IP injection with 1×10^7^ parasitized erythrocytes of PbANKA. Two hours after infection, they were given orally with 1, 10, 50, and 100 mg/kg of allicin once a day for 4 consecutive days (D0-D3). Positive control was also performed using ART (1, 5, 10, and 20 mg/kg), while the untreated control was given 10 ml/kg of 20% Tween-80. On D4, parasitemia was estimated, and effective dose 50 (ED_50_) values of allicin and ART were subsequently estimated. Percentage of inhibition was then calculated using the following formula.

(2) $$\begin{array}{c} \text{\%inhibition}=\frac{\left(\text{Parasitemia if untreated group}-\text{parasitemia if treated group}\right)}{\text{Parasitemia if untreated group}}\times 100\text{.} \end{array}$$

### 2.5. Combination Treatment of Allicin and ART against PbANKA-Infected Mice

For combination assay between allicin and ART in mice infected with PbANKA, the standard 4-day suppressive test was carried out [16]. PbANKA-infected Balb/c mice were divided into 7 groups (5 mice each) and given orally with combination at the doses of their respective ED_50_ and fixed combination ratio (1 : 1) of their respective ED_50_ of 1/2, 1/4, and 1/8 once a day for 4 consecutive days. The doses at ED_50_ of allicin and ART were also performed. On D4, parasitemia and % inhibition were calculated.

### 2.6. Packed Cell Volume Measurement

In order to evaluate the protective effect of the test compound on malaria-induced hemolysis, packed cell volume (PCV) was carried out. Tail blood from each mouse was collected in heparinized capillary tubes and sealed with cray. After centrifugation at 12000 rpm for 15 min, PCV was subsequently measured using the following formula on D0 and D4.

(3) $$\begin{array}{c} \text{PCV}=\frac{\text{Volume of erythrocytes in a given volume of blood}}{\text{Total blood volume}}\times 100\text{.} \end{array}$$

### 2.7. Bodyweight and Rectal Temperature Determination

The bodyweight (BW) was recorded with a sensitive digital balance on D0 and D4 of each mouse in all groups. Additionally, in order to investigate the protective effect on reduction of body temperature in each mouse, rectal temperature was measured using a digital thermometer.

### 2.8. Mean Survival Time

Any death of mice was recorded within 30 days of the study period to investigate allicin and its combination for improvement in survival days. The calculation of mean survival time (MST) was done using the following formula.

(4) $$\begin{array}{c} \text{MST}=\frac{\text{Sum of survival time of all mice in a group}}{\text{Total number of mice in that group}}\text{.} \end{array}$$

### 2.9. Statistical Analysis

Data were presented as mean and SEM (standard error of mean) using the commercial program GraphPad Prism (GraphPad Software version 6.05, Inc., USA). The best-fit ED_50_ value was calculated by the nonlinear regression function for sigmoidal dose-response variable slope. The comparison between the mean of measured parameters (parasitemia, PCV, BW, rectal temperature, and MST) was analyzed with one-way ANOVA with Tukey's post-hoc test. Statistical significance was considered at 95% confidence, *P* < 0.05. Additionally, combination index (CI) was simulated automatically by CompuSyn software (CompuSyn, Inc., USA). Synergism, additive effect, and antagonism were defined as CI < 1.0, CI = 1.0, and CI > 1.0, respectively.

## 3. Results

### 3.1. Antimalarial Activity of Allicin and ART against PbANKA Infection in Mice

**Figure 1:**
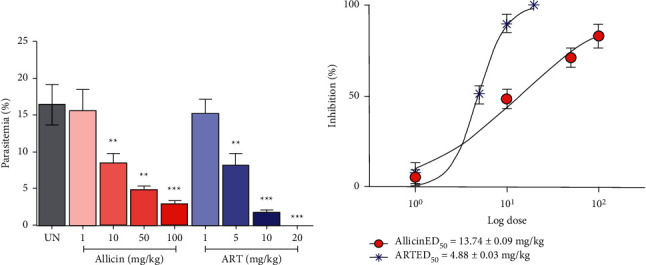
Effect of allicin and ART on PbANKA infection in mice. Groups of Balb/c mice were inoculated with 1×10^7^ parasitized erythrocytes of PbANKA by IP injection and given orally with allicin (1, 10, 50, and 100 mg/kg) and ART (1, 5, 10, and 20 mg/kg) for 4 consecutive days. (a) Parasitemia and % inhibition were calculated, and (b) dose-response curves of the antimalarial effect of (•) allicin and (^*∗*^) ART were also investigated. UN, untreated control. ^*∗*^*P* < 0.05, ^*∗∗*^*P* < 0.01, and ^*∗∗∗*^*P* < 0.001 compared to UN.

Allicin revealed dose-dependent antimalarial activity against PbANKA-infected mice. The analysis of parasitemia on day 4 revealed significant (*P* < 0.01) PbANKA suppression at the doses of 10, 50, and 100 mg/kg compared to the untreated group with 48.41%, 70.98%, and 82.93% inhibition, respectively (Figure 1(a)). However, at the dose of 1 mg/kg of allicin, no significant suppression was observed. Moreover, the mice treated with ART at 5, 10, and 20 mg/kg doses revealed significant (*P* < 0.01) inhibition of 50.98%, 89.76%, and 100%, respectively, compared to the untreated group. ART at a dose of 20 mg/kg treated mice and was free of any parasitemia on day 4. Additionally, the ED_50_ values of allicin and ART from the dose-response curves were 13.74 + 0.09 mg/kg and 4.88 + 0.03 mg/kg, respectively (Figure 1(b)).

### 3.2. Effect of Combination Treatment on Parasitemia in PbANKA Infection in Mice

**Figure 2:**
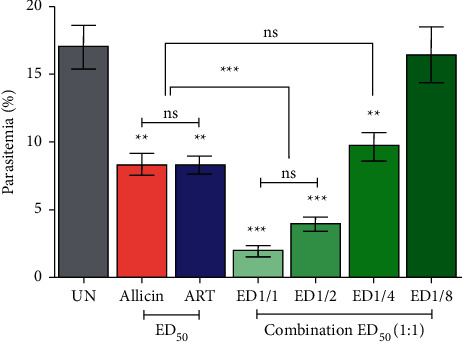
Effect of combination treatment on PbANKA infection in mice. Groups of Balb/c mice were inoculated with 1×10^7^ parasitized erythrocytes of PbANKA by IP injection and given orally with combination treatment between allicin and ART at the doses of their respective ED_50_ and fixed ratio (1 : 1) of their respective ED50 of 1/2, 1/4, and 1/8 for 4 consecutive days. Parasitemia was estimated. UN, untreated control. ^*∗∗*^*P* < 0.01 and ^*∗∗∗*^*P* < 0.001 compared to UN.

As shown in Figure 2, combination treatment (1 : 1) of allicin and ART at ED_50_ 1/1, ED_50_ 1/2, and ED_50_ 1/4 revealed significant (*P* < 0.01) antimalarial activity compared to the untreated group with inhibition of 88.82%, 76.94%, and 43.18%, respectively. Interestingly, ED_50_ 1/1 and ED_50_ 1/2 showed more potent antimalarial activity with significant (*P* < 0.001) than single treatment with allicin and ART. Additionally, the combination treatment showed synergistic interaction at levels of ED_50_ 1/1 (CI = 0.60925), ED_50_ 1/2 (CI = 0.65545), and ED_50_ 1/4 (CI = 0.73132) as indicated by the CI value less than 1.0 (Table 1). However, ED_50_ 1/8 of allicin and ART combination showed antagonistic interaction (CI = 4.11042).

### 3.3. Effects of Allicin and its Combination with ART on PCV, BW, Rectal Temperature, and MST in PbANKA Infection in Mice

Allicin and its combination with ART at the doses of ED_50_ 1/1, ED_50_ 1/2, and ED_50_ 1/4 exerted significant (*P* < 0.01) prevention of the PCV reduction (Table 2). Measurement of BW of infected mice also showed that allicin and its combination had no BW loss significantly (*P* < 0.01) compared to untreated control (Table 3). Moreover, allicin and its combination with ART significantly (*P* < 0.01) prevented rapid dropping of rectal temperature in PbANKA-infected mice (Table 4). Additionally, significant (*P* < 0.05) prolonged MST was observed in mice treated with allicin and its combination (Table 5). Interestingly, combination at ED_50_ 1/1 and ED_50_ 1/2 presented significant effects compared with the untreated group and single treatment. However, the combination at a dose of ED_50_ 1/8 failed to inhibit PCV reduction, BW loss, and rapid dropping of rectal temperature and did not present prolonged MST.

## 4. Discussion

The antimalarial activity of allicin and its combination with ART against PbANKA-infected mice in the 4-day suppressive test model was reported in this study. The 4-day suppressive test is a standardized assay commonly used for screening of antimalarial compounds in mice. The tested compounds resulting >30% inhibition of parasitemia are considered as active [17]. Accordingly, allicin which showed 48.41%, 70.98%, and 82.93% inhibition at 10, 50, and 100 mg/kg, respectively, can be classified as active. The inhibition of parasitemia of allicin-treated mice in the present study agreed with the previous report which showed significantly decreased parasitemia by allicin at a dose of 9 mg/kg [15]. Therefore, the inhibition of parasitemia of allicin-treated mice changed significantly from those in the untreated control showing that allicin has antimalarial activity, supporting the folk use of this compound as an antimalarial herb. The possible mechanism of antimalarial activity of allicin might be through the antioxidant effect, free radical scavenging, interference with protein synthesis, inhibition of erythrocyte invasion by parasites, or any unknown mechanisms [18]. It has been reported that allicin inhibited proteolytic cleavage of circumsporozoite protein and cell invasion and exerted the antimalarial effect on erythrocytic stage of parasite by inhibition of cysteine protease [19]. *Plasmodium* cysteine protease is required for parasite growth in the erythrocytic stage, and inhibition of this enzyme can decrease parasitemia [20]. Therefore, it can be suggested that the antimalarial effect of allicin might be due to the presence of parasite cysteine protease inhibition. It is also possible that allicin might change the host's immune response and thereby alter the outcome of the infection [21].

Combining allicin with ART (1 : 1) at the doses of ED_50_ 1/1, ED_50_ 1/2, and ED_50_ 1/4 showed significant antimalarial activity in comparison to the untreated control and the use of only allicin or ART. A good synergistic effect was also observed, as confirmed by the CI value less than 1. The mechanisms involved in the enhanced antimalarial activity observed with allicin and ART combination have not yet been elucidated. However, this could be attributed to the two drugs interacting with different targets in the parasite. Allicin might inhibit *Plasmodium* cysteine protease, an enzyme required for parasite growth in the erythrocytic stage, whilst ART is believed to activate the peroxide group in the presence of ferrous ion to form a carbon-centered radical which alkylates vital parasite proteins [5, 19]. Hence, the inhibition of different metabolic steps in parasites might contribute to the enhanced antimalarial activity of allicin and ART. However, its lower dose of combination (ED_50_ 1/8) did not even significantly affect parasitemia. On this basis, it is strongly believed that the combination of allicin and ART is an alternative antimalarial combination development.

In the treatment of malaria, it is crucial to pay attention to the inhibition of parasite growth and the reduction of the symptoms of the infection, which independently increases the pathogen burden. The discovery of a new antimalarial drug is expected to prevent PCV reduction and BW loss and decrease rectal temperature due to malaria infection [22]. PCV was measured to assess allicin's effectiveness and its combination with ART in preventing hemolysis due to increasing parasitemia. The major causes of severe anemia in PbANKA-infected mice include destruction of infected erythrocytes caused by the multiplication of parasites or by the activity of reticuloendothelial cells in the spleen, increased erythrocyte fragility, suppression of erythropoietin, and dyserythropoiesis [23–27]. A significant reduction of PCV was observed in the untreated mice which increased day to day until animal death. In the present study, allicin and its combination with ART reversed the PCV reduction indicating the antihemolysis. This could be due to the antimalarial activity or sustaining the availability of new erythrocytes produced in the bone marrow [24].

BW loss prevention is also another parameter to confirm the antimalarial activity of new natural or synthetic antimalarial drugs as BW loss is a characteristic of PbANKA-infected mice resulting from appetite loss, hypoglycemic effect, and metabolic disturbance of the parasite [28]. In this study, allicin and its combination with ART prevented BW loss in a dose-dependent manner. It might be due to the antimalarial effect, appetite enhancing, or immunomodulation [9]. In the case of PbANKA-infected mice, malaria is associated with hypothermia due to a reduction in internal body temperature and metabolic rates [22]. In this experiment, allicin and its combination with ART were able to prevent rectal temperature reduction compared to untreated control significantly. This could be attributed to the antimalarial effect of these compounds.

MST is another parameter that evaluates the antimalarial activity of tested compounds. Accordingly, a tested compound that can prolong the MST of infected mice compared to the untreated controls is considered an active antimalarial agent [29]. In this study, the infected mice treated with allicin and its combination with ART had significantly lived longer than the untreated control, and this might be due to the antimalarial activity of these compound. Hence, allicin and its combination with ART are active for antimalarial effects against PbANKA-infected mice.

## 5. Conclusion

There is an urgent need to develop of new antimalarial compounds to meet the challenges of antimalarial drug resistance. In this study, allicin combined with ART exerted potent antimalarial activity with a synergistic effect against PbANKA-infected mice. Moreover, protective effects of combination treatment on PCV reduction, rectal temperature reduction, and BW loss were observed with significantly prolonged MST. This study, for the first time, scientifically validates the traditional claim of allicin and its combination with ART for their antimalarial property. However, the small size of the sample might be a limitation of the study, and another mechanism of action against malaria is recommended.

## Figures and Tables

**Table 1:** Combination index of combination between allicin and ART against PbANKA infection in mice.

Test	Dose (mg/kg)			CI value
Combination (1 : 1)		Allicin	ART	
	ED_50_	14	5	0.60925^a^
	ED_50_ 1/2	7	2.5	0.65545^a^
	ED_50_ 1/4	3.5	1.25	0.73132^a^
	ED_50_ 1/8	1.75	0.625	4.11042^b^

^a^CI < 1, synergism; ^b^CI > 1, antagonism.

**Table 2:** Effect of allicin and its combination with ART on PCV in PbANKA infection in mice.

Group	Dose (mg/kg)			PCV (%)		
	UN	Allicin	ART	D0	D4	% change
Single	10 ml/kg of 20% Tween-80	—	—	53.32 + 0.85	41.92 + 1.21	−21.38
	—	14	—	52.78 + 1.21	46.26 + 0.69	−12.35^*∗*^
	—	—	5	53.19 + 0.98	47.72 + 0.58	−10.28^*∗*^
Combination (1 : 1)	ED_50_	14	5	52.62 + 0.73	51.74 + 0.95	−1.67^*∗∗*^^,##^
	ED_50_ 1/2	7	2.5	53.54 + 0.87	51.83 + 1.03	−3.19^*∗∗*^^,##^
	ED_50_ 1/4	3.5	1.25	52.29 + 0.61	46.48 + 0.63	−11.11^*∗*^
	ED_50_ 1/8	1.75	0.625	53.29 + 0.81	42.15 + 1.13	−20.90

^*∗*^ *P* < 0.05 and ^*∗∗*^*P* < 0.01 compared to UN. ^##^*P* < 0.01 compared to allicin and ART.

**Table 3:** Effect of allicin and its combination with ART on BW in PbANKA infection in mice.

Group	Dose (mg/kg)			BW (g)		
	UN	Allicin	ART	D0	D4	% change
Single	10 ml/kg of 20% Tween-80	—	—	26.51 + 1.13	20.05 + 0.72	−24.37
	—	14	—	27.12 + 0.52	25.58 + 0.26	−5.68^*∗*^
	—	—	5	26.34 + 1.23	25.19 + 0.82	−4.37^*∗*^
Combination (1:1)	ED_50_	14	5	26.47 + 0.86	26.53 + 0.34	0.23^*∗∗*^^,##^
	ED_50_ 1/2	7	2.5	26.71 + 0.91	26.65 + 0.63	−0.22^*∗∗*^^,##^
	ED_50_ 1/4	3.5	1.25	25.59 + 1.06	24.91 + 0.93	−2.66^*∗*^
	ED_50_ 1/8	1.75	0.625	26.69 + 0.93	20.17 + 0.51	−24.43

^*∗*^ *P* < 0.05 and ^*∗∗*^*P* < 0.01 compared to UN. ^##^*P* < 0.01 compared to allicin and ART.

**Table 4:** Effect of allicin and its combination with ART on rectal temperature in PbANKA infection in mice.

Group	Dose (mg/kg)			Rectal temperature (°C)		
	UN	Allicin	ART	D0	D4	% change
Single	10 ml/kg of 20% Tween-80	—	—	36.40 + 0.14	34.42 + 0.26	−5.44
	—	14	—	36.22 + 0.21	35.91 + 0.17	−0.86^*∗*^
	—	—	5	36.67 + 0.18	36.22 + 0.23	−1.23^*∗*^
Combination (1 : 1)	ED_50_	14	5	36.18 + 0.24	36.65 + 0.18	1.30^*∗∗*^^,##^
	ED_50_ 1/2	7	2.5	36.53 + 0.29	36.55 + 0.29	0.05^*∗∗*^^,##^
	ED_50_ 1/4	3.5	1.25	36.02 + 0.34	35.58 + 0.25	−1.22^*∗*^
	ED_50_ 1/8	1.75	0.625	36.90 + 0.27	34.21 + 0.45	−7.29

^*∗*^ *P* < 0.05 and ^*∗∗*^*P* < 0.01 compared to UN. ^##^*P* < 0.01 compared to allicin and ART.

**Table 5:** Effect of allicin and its combination with ART on MST in PbANKA infection in mice.

Group	Dose (mg/kg)			MST (day)
	UN	Allicin	ART	
Single	10 ml/kg of 20% Tween-80	—	—	10.3 + 1.2
	—	14	—	17.2 + 1.4^*∗*^
	—	—	5	22.4 + 1.1^*∗*^
Combination (1 : 1)	ED_50_	14	5	30.0 + 0.0^*∗∗*^
	ED_50_ 1/2	7	2.5	30.0 + 0.0^*∗∗*^
	ED_50_ 1/4	3.5	1.25	23.5 + 1.2^*∗*^
	ED_50_ 1/8	1.75	0.625	11.4 + 1.5

^*∗*^ *P* < 0.05 and ^*∗∗*^*P* < 0.01 compared to UN.

## Data Availability

The data used to support the findings of this study are deposited in the figshare.com repository (https://figshare.com/s/67713cf58ab40dcca2f0. DOI: 10.6084/m9.figshare.14450151).
